# Role of Reactive Oxygen Species in Glucose Metabolism Disorder in Diabetic Pancreatic β-Cells

**DOI:** 10.3390/biom12091228

**Published:** 2022-09-02

**Authors:** Eri Mukai, Shimpei Fujimoto, Nobuya Inagaki

**Affiliations:** 1 Medical Physiology and Metabolism Laboratory, Department of Biomedical Sciences, College of Life Sciences, Ritsumeikan University, Kusatsu 5258577, Japan; 2 Department of Endocrinology, Metabolism, and Nephrology, Kochi Medical School, Kochi University, Kochi 7838505, Japan; 3 Department of Diabetes, Endocrinology and Nutrition, Graduate School of Medicine, Kyoto University, Kyoto 6068507, Japan

**Keywords:** reactive oxygen species, pancreatic β-cells, insulin secretion, glucose metabolism, oxidative stress

## Abstract

The dysfunction of pancreatic β-cells plays a central role in the onset and progression of type 2 diabetes mellitus (T2DM). Insulin secretory defects in β-cells are characterized by a selective impairment of glucose stimulation, and a reduction in glucose-induced ATP production, which is essential for insulin secretion. High glucose metabolism for insulin secretion generates reactive oxygen species (ROS) in mitochondria. In addition, the expression of antioxidant enzymes is very low in β-cells. Therefore, β-cells are easily exposed to oxidative stress. In islet studies using a nonobese T2DM animal model that exhibits selective impairment of glucose-induced insulin secretion (GSIS), quenching ROS generated by glucose stimulation and accumulated under glucose toxicity can improve impaired GSIS. Acute ROS generation and toxicity cause glucose metabolism disorders through different molecular mechanisms. Nuclear factor erythroid 2-related factor 2 (Nrf2), a transcription factor, is a master regulator of antioxidant defense and a potential therapeutic target in oxidative stress-related diseases, suggesting the possible involvement of Nrf2 in β-cell dysfunction caused by ROS. In this review, we describe the mechanisms of insulin secretory defects induced by oxidative stress in diabetic β-cells.

## 1. Introduction

Type 2 diabetes mellitus (T2DM), a metabolic disorder characterized by chronic hyperglycemia, is caused by relative insulin deficiency. An increase in insulin demand induced by insulin resistance in peripheral tissues, including the liver, muscle, and adipose tissue, exceeds the capacity for insulin secretion in pancreatic β-cells. The subsequent failure of pancreatic β-cells, which is attributed to the loss of their mass and functional deterioration, is essential for the onset and progression of T2DM [1,2,3]. Previous studies using specimens from autopsies or surgical operations have shown that β-cell mass is 30~41% lower in T2DM subjects than in normal, lean subjects (and 63% lower in obese T2DM subjects than in nondiabetic obese subjects) [4,5]. Furthermore, subsequent examination revealed that β-cell mass was 24 and 54% lower in subjects with <5 and >15 years of clinical diabetes, respectively, indicating that decreases in β-cell mass correspond to an increase in the duration of T2DM. These findings suggest that a small difference in β-cell mass observed within 5 years of onset is insufficient to cause T2DM, and that the decrease in mass with the increased duration of the disease can be a consequence of its progression [6]. Moreover, the possibility of overestimation of β-cell loss in T2DM has been reported [7]. Therefore, β-cell dysfunction likely plays a central role in disease progression. Pancreatic β-cells are metabolically active tissues, and ATP produced by glucose metabolism within the cells is essential for the exocytosis of insulin granules [8,9]. Previous studies using rodent and human islets have shown impaired glucose-stimulated insulin secretion (GSIS) and ATP production in T2DM [8]. A high level of glucose metabolism for insulin secretion generates reactive oxygen species (ROS) via the mitochondrial respiratory chain. Additionally, low levels of antioxidant enzymes in β-cells make them susceptible to oxidative stress-induced damage [10]. In this review, we discuss the role of ROS in glucose metabolism disorders that induce impaired insulin secretion in diabetic pancreatic β-cells.

## 2. Mechanism of Glucose-Stimulated Insulin Secretion in β-Cells

**Figure 1:**
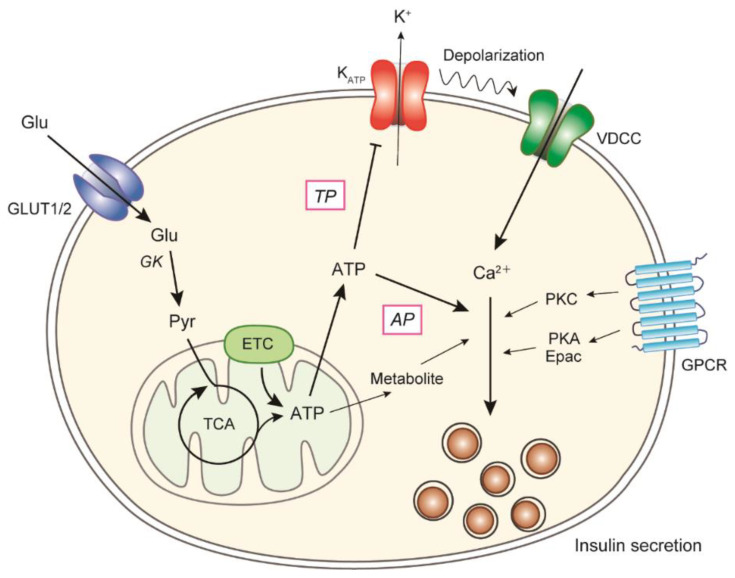
Mechanism of glucose-induced insulin secretion in β-cells. Insulin secretion from β-cells is regulated by intracellular glucose metabolism, and glucose-induced ATP production in mitochondria plays an essential role. Increased ATP levels in β-cells lead to the closure of K_ATP_ channels, followed by membrane depolarization and the subsequent activation of VDCCs. An elevation of intracellular Ca^2+^ levels triggers the exocytosis of insulin granules. The triggering pathway is critical for GSIS and is modulated by the signals of various GPCRs. In addition, ATP and other metabolites amplify downstream of Ca^2+^ influx in the triggering pathway (the amplifying pathway). Glu, glucose; GK, glucokinase; Pyr, pyruvate; ETC, electron transport chain; TP, the triggering pathway; AP, the amplifying pathway.

Glucose is the most important secretagogue for insulin, and the molecular mechanism of GSIS is shown in Figure 1. Glucose enters β-cells through glucose transporters (GLUT2 in rodents and GLUT1 in humans [11,12]) and is phosphorylated by glucokinase [13]. Glucose uptake is not rate-limiting because of the large capacity of transporters. Mutations in the human glucokinase gene cause the development of an autosomal dominant form of diabetes (maturity-onset diabetes of the young 2; MODY2) [14], therefore indicating the importance of glucokinase in the insulin secretory mechanism and as a glucose sensor in β-cells. Increases in glycolysis flux and the activity of the tricarboxylic acid (TCA) cycle lead to an increase in mitochondrial ATP production. The increased ATP/ADP ratio within the cytoplasm promotes the closure of ATP-sensitive K^+^ (K_ATP_) channels that are composed of subunits of the potassium channel (Kir6.2) and sulfonylurea receptor 1 (SUR1) [15,16], followed by an attenuation of plasma membrane polarization. Membrane depolarization leads to the activation of voltage-dependent Ca^2+^ channels (VDCCs), and the influx of extracellular Ca^2+^. The resultant increase in intracellular Ca^2+^ levels triggers the exocytosis of insulin-containing secretory granules [8,9,17]. This pathway is critical for GSIS and is modulated by many factors, including hormones, neurotransmitters, and nutrients, which activate PKA and PKC via their specific G protein-coupled receptors (GPCRs) to act around the site of Ca^2+^ influx [18,19]. In addition to the classical mechanism (the triggering pathway), the glucose amplification pathway plays a role in GSIS [17]. The amplifying pathway was originally identified by pharmacological studies involving the clamping of the Ca^2+^ concentration within β-cells at a high level [20,21]. Under these conditions, an increase in glucose levels augments insulin secretion, even though glucose does not increase the Ca^2+^ concentration. These findings indicate that glucose produces a signal for insulin secretion other than increasing intracellular Ca^2+^. The metabolic amplifying pathway is physiologically and quantitatively important; however, the molecular mechanisms have not been completely understood [3,22], although it is known that ATP derived from glucose metabolism is involved [23,24,25].

The anaplerotic metabolism of glucose has been implicated in producing a source of coupling factors for GSIS [3]. The anaplerotic metabolism of pyruvate and other fuels leads to an increase in the levels of TCA cycle intermediates, followed by the export of mitochondrial intermediates to the cytosol and their subsequent engagement in cytosolic reactions. Recent studies have focused on the pyruvate–isocitrate cycle, in which the anaplerotic entry of pyruvate into the TCA cycle via pyruvate carboxylase (not mediated by acetyl-CoA) provides mitochondrial citrate and isocitrate [26,27]. Increased cytosolic isocitrate through the mitochondrial citrate/isocitrate carrier [28] engages with cytosolic NADP^+^-dependent isocitrate dehydrogenase 1 (IDH1) to generate 2-ketoglutarate and NADPH [29]. Cytosolic NADPH produced by the IDH1 reaction is linked to glutaredoxin-mediated activation of sentrin/SUMO-specific protease-1 (SENP1), a de-SUMOylase enzyme that activates the exocytosis of insulin granules [30].

The glycerol shunt operated by glycerol-3-phosphate phosphatase (G3PP) regulates β-cell metabolism and GSIS [31]. G3PP dephosphorylates glycerol-3-phosphate to form glycerol; glycerol-3-phosphate is produced from glucose via glycolysis and from lipolysis-derived glycerol by glycerol kinase. In islets from β-cell-specific G3PP deletion mice, ATP production and insulin secretion are enhanced at high glucose levels, suggesting that the glycerol shunt plays a role in preventing insulin hypersecretion in hyperglycemia [31].

Pyruvate kinase (PK) is an ATP/ADP generator that regulates the closure of K_ATP_ channels to stimulate insulin secretion [32]. PK converts ADP and phosphoenolpyruvate from glycolysis and mitochondrial anaplerosis into ATP and pyruvate. Glucose entry into β-cells lowers ADP through PK to levels sufficient to close K_ATP_ channels, although the flux of the TCA cycle is low. Following membrane depolarization, the level of ADP is restored, and the flux of the TCA cycle is increased, inducing mitochondrial oxidative phosphorylation-dependent ATP production. Therefore, glucose metabolism is spatially and temporally compartmentalized. Future studies focusing on all aspects of glucose metabolism, including unknown pathways other than the current consensus model, are warranted.

## 3. ROS Generation and Antioxidant Defense in β-Cells

ROS are a group of reactive molecules and free radicals derived from oxygen. The reduction of oxygen by a single electron generates a superoxide anion (O_2_^−^) that is the precursor of most forms of ROS. O_2_^−^ is rapidly converted into hydrogen peroxide (H_2_O_2_) by superoxide dismutase (SOD). H_2_O_2_ is fully reduced to water by glutathione peroxidase (Gpx) or catalase. In the presence of high concentrations of transition metals, such as Cu^2+^ or Fe^2+^, H_2_O_2_ is reduced to hydroxyl radicals, one of the strongest oxidants in nature [33]. The mitochondrial electron transport chain is a major source of ROS because electron leakage occurs mainly in complexes I and III. O_2_^−^ is one of the most abundant but short-lived ROS [34], and up to 4% of the total oxygen consumed is converted into O_2_^−^ during oxidative phosphorylation [35]. In β-cells, a rapid increase in glycolytic flux is tightly coupled with the activation of mitochondrial oxidative phosphorylation [36], and almost 100% of the carbon in glucose is oxidized to CO_2_ [37]. Therefore, the high level of glucose metabolism in β-cells frequently generates ROS in mitochondria. Additionally, the expression of SOD and Gpx/catalase in β-cells is approximately 30 and 5% of the liver values, respectively [10]. Owing to the low expression of antioxidant enzymes, β-cells are susceptible to damage from oxidative stress. Since exposure of β-cells to high glucose induces mitochondrial ROS generation [38,39], the insulin secretory function of β-cells may be impaired.

An increase in intracellular Ca^2+^ induced by glucose metabolism activates protein kinase C and subsequently NADPH oxidase (NOX) [40]. The NOX family is a group of plasma or subcellular membrane-associated enzymes, which generate O_2_^−^. β-cells express NOX2 (prototypical NOX), which is composed of gp91phox (defining the NOX isoform) and p22phox, located in the plasma membrane, and cytosolic components of the NADPH complex (p47phox, p67phox, p40phox, and small G-protein Rac 1/2) [41,42]. NOX inhibition disrupts intracellular Ca^2+^ dynamics and impairs GSIS [43,44]. Therefore, NOX activation contributes to ROS generation during GSIS [42], and ROS can act as metabolic signaling molecules for GSIS [45,46]. In contrast, experiments using islets of *Nox2* knockout mice showed that NOX2 generates O_2_^−^ to reduce GSIS [47], and that NOX2 is involved in impaired insulin secretion by cytokines [48]. In islets of Zucker diabetic fatty rats, the expression of NOX2 components was higher than that in islets of lean control rats [49]. However, de Souza et al. indicated that NOX2 does not contribute to glucose-induced oxidative stress or alterations in β-cell function [50]. Therefore, the role of NOX in β-cell metabolism and function remains to be elucidated.

Peroxynitrite (ONOO^−^) is a reactive species generated by O_2_^−^ and free radical nitric oxide (NO). It has been suggested that ONOO^−^ contributes to β-cell damage in response to pro-inflammatory cytokines [51,52]. However, β-cells do not generate ONOO^−^ by cytokine stimulation [53]. When β-cells are forced to generate ONOO^−^ by providing exogenous O_2_^−^ and NO, O_2_^−^ scavenges NO to form ONOO^−^, resulting in the attenuation of NO-induced damage [53]. These findings suggest that NO is a toxic mediator of cytokines, and ONOO^−^ generation protects β-cells from NO-induced damage.

Peroxiredoxins represent a family of antioxidant proteins, and six members of the peroxiredoxin family have been identified. Peroxiredoxin catalyzes the reduction of H_2_O_2_ to water, and oxidized peroxiredoxin is reduced by thioredoxin that, in turn, becomes oxidized. Oxidized thioredoxin is reduced by thioredoxin reductase using NADPH [54]. Peroxiredoxin and thioredoxin are important for redox signaling and are part of the first line of defense against H_2_O_2_. The expression of peroxiredoxins (1 and 2) in the cytoplasm of β-cells is upregulated by stressors, including H_2_O_2_, cytokines, streptozotocin, and alloxan [55]. Peroxiredoxin 3, a mitochondrial isoform, is also expressed in β-cells, and its expression is increased by cytokine stimulation [56]. β-cells also possess peroxiredoxin 4, an ER-specific isoform [57]. Overexpression of these peroxiredoxins reduces the damage of β-cells caused by several stressors [56,57,58]. In contrast, overexpression of thioredoxin in β-cells suppresses the progression of hyperglycemia in db/db mice, a diabetic model with obesity [59]. Interestingly, cytosolic thioredoxin 1 is secreted from β-cells by hypoxia and glucose stimulation, and exogenously added thioredoxin 1 improves impaired insulin secretion by hypoxia, suggesting paracrine regulation of β-cell function by thioredoxin [60]. Thioredoxin-interacting protein (TXNIP), identified as thioredoxin-binding protein-2, which is identical to vitamin D3 upregulated protein 1 [61], was found to be the most strongly upregulated gene in response to glucose in human islets [62]. TXNIP binds and inhibits thioredoxin, thereby inducing oxidative stress by modulating the cellular redox state [63]. TXNIP expression in β-cells is elevated in diabetic rodent models with and without obesity, and TXNIP deficiency protects β-cells from death [63]. Surprisingly, β-cells remove micromolar levels of H_2_O_2_ via the peroxiredoxin/thioredoxin antioxidant system [64]. Therefore, the peroxiredoxin/thioredoxin antioxidant system may compensate for inadequate antioxidant capacity owing to the low expression of antioxidant enzymes in β-cells.

## 4. Contribution of Acute ROS Generation by Glucose to Diabetic β-Cell Dysfunction

In T2DM, the characteristics of insulin secretory defects in β-cells include a selective impairment of glucose stimulation, while insulin secretion stimulated by arginine, a membrane-depolarizing agent, is preserved [8,65]. Our colleagues and other investigators have demonstrated the mechanism of impaired GSIS in T2DM animal models. The Goto-Kakizaki (GK) rat, an inbred polygenic model of nonobese T2DM, exhibits valuable characteristics that are functionally present in human diabetic patients [66,67,68]. Selective impairment of GSIS in GK rats has been demonstrated in vivo and in isolated pancreatic islets [69,70,71]. Studies on islet cells of GK rats have revealed that the responses to glucose are impaired, while the functions of K_ATP_ channels and VDCCs are maintained [72,73,74]. ATP production by glucose stimulation is lowered in the islets of GK rats [75], which parallels the impairment of glucose-induced ATP production in the islets of human patients with T2DM [76]. The glycerol phosphate shuttle is the metabolic site responsible for the impaired ATP production because of a low activity of mitochondrial glycerol phosphate dehydrogenase (mGPDH) [77,78,79]. However, mGPDH overexpression does not correct GSIS in the islets of GK rats [80]. Glucose utilization (the glycolytic rate) and glucose oxidation (the rate of the TCA cycle) have been reported to be decreased, unchanged, or even enhanced in the islets of GK rats [70,75,81], while these are decreased in islets of human T2DM patients [78,82]. These contradictory findings may be at least partly attributed to the differences between various GK rat colonies. Additionally, factors that impair glucose metabolism, rather than the failure of specific sites, may contribute to low ATP production.

**Figure 2:**
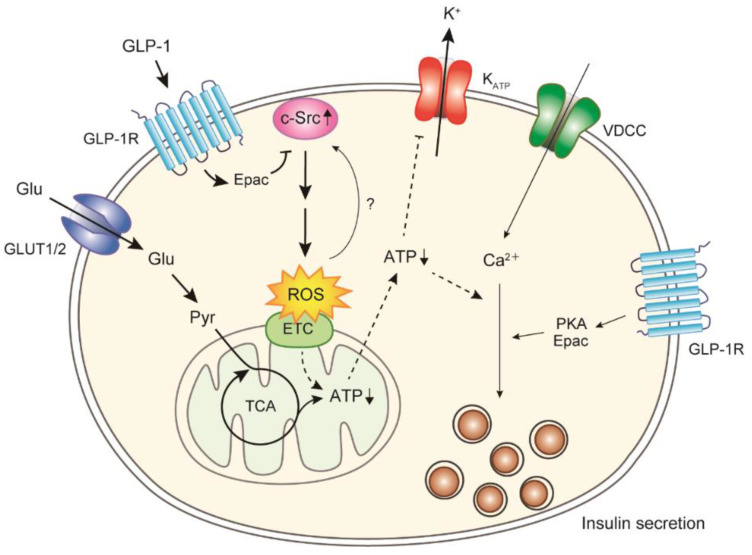
Involvement of c-Src in glucose-induced ROS generation in diabetic β-cells. In diabetic β-cells, acute ROS generation induced by glucose is upregulated, which is attributed to c-Src activation. GLP-1 signaling can suppress c-Src activation and ROS generation, resulting in restored ATP production. Glu, glucose; Pyr, pyruvate; ETC, electron transport chain.

We revealed that glucose-induced ROS generation is accelerated in the islets of GK rats and that ROS scavengers (α-tocopherol plus ascorbate) restore the GSIS and ATP production in the islets of GK rats to those of normal Wistar rats [83]. Interestingly, abnormalities in ROS generation, ATP production, and insulin secretion caused by glucose are also restored by Src inhibitors [83,84]. The Src family kinases (SFKs) are a family of nonreceptor tyrosine kinases associated with the cell membrane, and they play important roles in various signal transductions [85]. The c-Src proto-oncogene, the oldest and most studied member of the SFKs, plays major roles in the development, growth, progression, and metastasis of numerous cancers [86]. The pancreatic islets express most SFKs similarly in Wistar and GK rats (undetectable level of Fyn), except for c-Src [84]. The level of c-Src pY416, which indicates Src activation, has been found to be higher in the islets of GK rats than in those of Wistar rats, and the levels of c-Src and carboxyl terminal Src kinase, a negative regulator of SFKs [85], have been found to be lower in the islets of GK rats than in those of Wistar rats [84], indicating that c-Src is activated under diabetic conditions and is degraded by ubiquitination [87] (Figure 2). Although the mechanism of c-Src activation under diabetic conditions is unclear, ROS themselves may regulate c-Src activity [88]. c-Src has also been reported to mediate the transactivation of tyrosine kinase receptors by GPCRs [89,90,91]. A few hundred GPCRs expressed in human and rodent islets mediate the regulation of insulin secretion by various substrates including hormones [18,19]. Glucagon-like peptide (GLP)-1 is an incretin peptide released from the intestine in response to nutrient ingestion, and it augments GSIS [92,93]. GLP-1 binds to the GLP-1 receptor (GLP-1R), a member of the GPCRs, and induces the elevation of cAMP levels via adenylyl cyclase and the subsequent activation of PKA- and/or Epac-dependent pathways to increase the exocytosis of insulin granules [92,93,94]. A GLP-1R agonist suppresses the phosphorylation of c-Src pY416, i.e., c-Src activation, and ameliorates excess ROS generation by glucose (without affecting antioxidant enzyme activities), resulting in restored ATP production in the islets of GK rats [84]. c-Src inactivation by GLP-1R stimulation is Epac-dependent and not PKA-dependent. The regulation of downstream proteins by Src signaling is complex; typical PI3K/Akt signaling [85] is one of the downstream pathways of c-Src regulating ROS generation [84]. Therefore, GLP-1 signaling can acutely suppress excessive ROS generation via c-Src activation in diabetic β-cells, in addition to other beneficial long-term effects, including the induction of β-cell proliferation and enhanced resistance to apoptosis [92,93] (Figure 2). In the islets of Nox2 knockout mice, Nox2 deficiency decreases ROS generation and increases cAMP formation. The increase in cAMP reduces ROS generation, indicating a mutual relationship between cAMP and ROS levels [47]. Therefore, Nox2 may be partially involved in ROS generation in diabetic β-cells. GLP-1R agonists and dipeptidyl peptidase-4 inhibitors, which delay GLP-1 degradation, are widely used in T2DM treatment [95]. Several clinical trials have shown that patients receiving combination therapy of a GLP-1R agonist and sulfonylurea (SU), a K_ATP_ inhibitor, have a higher incidence of hypoglycemia than those treated with a GLP-1R agonist only [96,97]. K_ATP_ channel inhibition by SU has been shown to be attenuated under conditions of decreased intracellular ATP in β-cells because of a defect in signal transduction between SUR1 and Kir6.2 [98]. Therefore, recovery of ATP production by GLP-1 signaling may improve the attenuated K_ATP_ channel inhibition by SU under diabetic conditions, resulting in an excessive drop in blood glucose levels.

## 5. ROS Toxicity and the Change in Glucose Metabolism in Diabetic β-Cells

**Figure 3:**
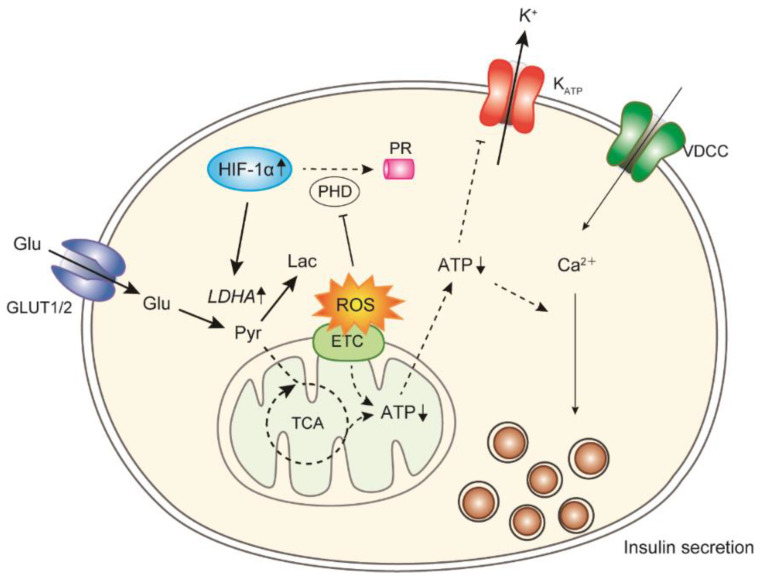
HIF-1α regulates the change in glucose metabolism by ROS toxicity in diabetic β-cells. Under chronic oxidative stress, ROS inhibits PHD activity to activate HIF-1α. LDHA activation by HIF-1α causes excess lactate production from glucose. Glu, glucose; Pyr, pyruvate; Lac, lactate; ETC, electron transport chain; PR, proteasome.

Oxidative stress is higher in the islets of both GK rats and human T2DM patients than in those of controls [4,99], and the elevation is dependent on the duration of chronic hyperglycemia [99]. Long-term exposure to high glucose causes impairment in insulin secretion and biosynthesis [100]. Prolonged oxidative stress activates stress-induced MAP kinases, such as JNK, suppressing the binding of transcriptional factors to the insulin promoter. Exposure of the islets of GK rats to cell-permeable antioxidant enzyme mimics (tempol plus ebselen) for 12 h leads to an ROS reduction, followed by an improvement in ATP production and insulin secretion [101]. Decreased glucose oxidation in GK rat islets is also elevated to levels observed in the islets of Wistar rats by treatment with antioxidant enzyme mimics. In addition, lactate production and the expression of lactate dehydrogenase A (LDHA), which converts pyruvate to lactate, are elevated in the islets of GK rats, and their elevations are reduced by treatment with antioxidant enzyme mimics. LDHA expression is upregulated in the islets of several T2DM model animals [102,103,104], and this LDHA overexpression is sufficient to disturb GSIS [105]. The expression of hypoxia-inducible factor-1α (HIF-1α), a potential upstream regulator of LDHA [106], is reduced by antioxidant treatment, and an HIF-1α inhibitor reduces lactate production and improves insulin secretion [101]. HIF-1 mediates adaptive responses to reduced O_2_ availability and regulates the balance between O_2_ supply and demand [106]. In properly oxygenated cells, HIF-1α is hydroxylated by prolyl hydroxylase domain (PHD) proteins using O_2_ and α-ketoglutarate, bound by von Hippel–Lindau (VHL) protein, ubiquitinylated, and degraded by the proteasome. In hypoxic cells, HIF-1α binds to HIF-1β, and the dimer induces the transcription of several genes, including pyruvate dehydrogenase (PDH) kinase 1 (PDK1), LDHA, and other genes of glycolytic enzymes. PDK1 phosphorylates and inactivates PDH, consequently inhibiting the conversion of pyruvate to acetyl-CoA for entry into the TCA cycle. Hypoxia occurs frequently in human cancers, which produce energy predominantly through accelerated glycolysis, resulting in lactate overproduction [106,107,108]. This metabolic phenomenon, known as aerobic glycolysis or the Warburg effect [109,110], can occur even under nonhypoxic conditions, indicating that HIF-1α is a central regulator of cancer metabolism. In addition, ROS inhibit PHD activity to stabilize HIF-1α under normoxia [111]. Therefore, ROS accumulation in diabetic β-cells may induce Warburg-like lactate production by HIF-1α as observed in cancer cells, resulting in the disruption of glucose sensing and insulin secretion [112] (Figure 3).

Mice with a β-cell-specific deletion of *VHL* exhibit increased HIF-1α expression and impaired GSIS [113,114,115]. In contrast, mice with an *HIF-1α* deletion in β-cells do not exhibit obvious changes in glucose response, while HIF-1α overexpression in β-cells impairs GSIS [113,114]. However, Cheng et al. reported glucose intolerance in mice with a β-cell-specific deletion of *HIF-1α*, as well as impaired GSIS in their islets and in β-cells with an HIF-1α deletion by RNAi [116]. Moreover, high-fat diet-induced glucose intolerance in mice was improved by HIF-1α activation. The expression of HIF-1α mRNA is decreased in the islets of T2DM subjects compared to that in people with normal glucose tolerance [117]. However, HIF-1α expression in the islets of GK rats does not change compared with that in Wistar rats [101]. These discrepancies may be attributed to differences in the duration of T2DM and the levels of hypoxia and ROS. The expression of HIF-1β mRNA is decreased in islets of T2DM subjects, and mice with a β-cell-specific *HIF-1β* deletion exhibit abnormal glucose tolerance and impaired insulin secretion [117]. These findings indicate that HIF-1 is undoubtedly involved in diabetic β-cell dysfunction, and an elucidation of the role of HIF-1 in T2DM may be helpful in identifying HIF-1 as a therapeutic target.

## 6. A Master Regulator of Antioxidant Defense: Nuclear Factor Erythroid 2-Related Factor 2 (Nrf2)

**Figure 4:**
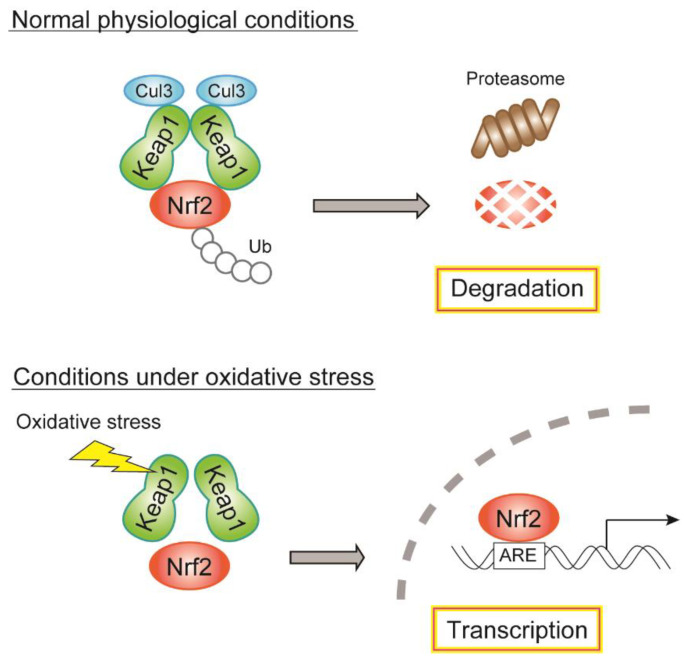
Regulatory mechanism of gene expression by the Nrf2/Keap1 system. During normal physiological conditions, Nrf2 binds to Keap1 and is degraded through Cul3-mediated ubiquitination. During oxidative stress, Nrf2 is released from the binding of Keap1, translocated to the nucleus, and drives gene expression of antioxidant and NADPH-producing enzymes. Cul3, Cullin3; Ub, ubiquitin.

Nrf2, isolated in 1994 [118], is a transcription factor that regulates over 100 genes related to oxidative stress and cell survival [119,120]. Under nonstressed conditions, Nrf2 binds to its cytosolic repressor, Kelch-like ECH-associated protein 1 (Keap1) [121], which serves as an adaptor for the Cullin3-based ubiquitin E3 ligase complex and is engaged in proteasomal degradation [122]. Under oxidative stress, cysteine residues on Keap1 are modified, which leads to the disruption of Keap1–Keap1 homodimerization, Keap1–Cullin3 interaction, and Nrf2 translocation into the nucleus. Several cysteine residues within Keap1, including Cys151, are thought to be the sites for oxidation-induced modification of ROS sensors [120]. Nrf2 binds to the promoter region of the antioxidant response element (ARE) and initiates the transcription of various cytoprotective enzymes, including SOD and Gpx, resulting in the restoration of redox balance in response to oxidative stress (Figure 4). Nrf2 regulates the basal and inducible expression of various genes of antioxidant enzymes, antioxidants, xenobiotic-metabolizing enzymes, and enzymes of the pentose phosphate pathway/NADPH production [120]. Therefore, the Nrf2/Keap1 system may maintain GSIS under diabetic conditions through the regulation of antioxidant-related genes as well as genes involved in NADPH production, which help in amplifying insulin secretion [3]. In addition, Nrf2 affects mitochondrial membrane potential and ATP synthesis [123,124]. Therefore, Nrf2 may be a prominent regulator of glucose metabolism in diabetic β-cells.

The mechanisms underlying alterations in the expression of Nrf2 and Keap1 in β-cells of T2DM patients are still unclear. The expression of Nrf2 mRNA in the islets is upregulated in streptozotocin-induced diabetic mice [125]. In contrast, exposure of β-cells to long-chain fatty acids (palmitate) for 48 h results in a decreased expression of Nrf2 mRNA (unpublished data). Since Nrf2 is translocated into the nucleus, further clarification regarding the change in Nrf2 levels in the nucleus under diabetic conditions is required. In a study using β-cell-specific *Keap1* deletion mice, Nrf2 activation reversed the reduced β-cell mass, impaired GSIS, and glucose intolerance induced by NO stress [126]. It has been reported that dimethyl fumarate and oltipraz, Nrf2 activators, antagonize glucotoxicity-induced impairment of insulin secretion, whereas GSIS under normal conditions is reduced by both Nrf2 activators [127]. Elevation of Nrf2 levels following exposure to low levels of arsenite increases antioxidant capacity and reduces GSIS in normal β-cells [128], suggesting that overactivation of antioxidant capacity disturbs the physiological redox balance [127]. Therefore, unnecessary Nrf2 activation may not be beneficial for nonstressed β-cells. Accumulating evidence suggests that the Nrf2/Keap1 pathway is implicated in diabetic oxidative damage to several peripheral tissues, and the pharmacological activation of Nrf2 is expected to be a promising therapeutic approach for T2DM [119,129,130]. The bardoxolone family, including 2-cyano-3,12-dioxo-oleana-1,9(11)-dien-28-oic acid (CDDO) methyl ester (CDDO-Me) and CDDO imidazolide (CDDO-Im), derivatives of synthetic triterpenoids, reduces blood glucose levels and improves insulin resistance in high-fat diet-fed and db/db mice [131,132]. Several natural compounds, including sulforaphane, curcumin, resveratrol, and quercetin, have been identified as Nrf2 activators, which exhibit antioxidative potential in various tissues [119,129,130]. Accordingly, the Nrf2/Keap1 axis is a promising pharmacological target for protecting β-cells from oxidative damage under diabetic conditions.

## 7. Conclusions

Glucose metabolism is crucial for insulin secretion in β-cells, and a highly activated glucose metabolism causes ROS generation, which impairs insulin secretion. This contradictory mechanism might be associated with the dual effects of ROS. Although ROS have negative effects on cellular function, it has been proposed that they contribute to the stimulation of GSIS [133]. Measurements of redox changes at the whole-cell level are limited in clarifying the role of ROS. Recent developments of specific redox probes targeting cellular compartments of interest may allow the identification of the site of ROS generation and the site impaired by ROS at the subcellular level [133]. The mechanisms of the regulation of redox signaling in response to glucose and ROS toxicity caused by glucose exposure are complex. Therefore, evaluation of the process in subcellular compartments will be helpful in clarifying the role of ROS in detail, and future studies using advanced techniques may lead to the identification of new methods to attenuate oxidative stress for diabetic therapy.

## Data Availability

Not applicable.
